# From feminine leisure to urban craze: the popularization of badminton in Republican Shanghai

**DOI:** 10.3389/fspor.2026.1717411

**Published:** 2026-05-18

**Authors:** Zhuomiao Zhao, Chang Liu, Kohei Kawashima

**Affiliations:** 1 Graduate School of Sport Sciences, Waseda University, Tokyo, Japan; 2 Faculty of Physical Education, International Budo University, Chiba, Japan; 3 Faculty of Sport Sciences, Waseda University, Tokyo, Japan

**Keywords:** badminton, gender, mixed-gender sports, Republican Shanghai, sports popularization

## Abstract

This paper investigates the gendered popularization of badminton in semi-colonial and wartime Shanghai between 1912 and 1949, focusing on how the sport moved from expatriate enclaves into local society, how women entered and were represented within this process, and how its inherited gender meanings were reworked in China. This article is a historical study based on newspapers and other primary archival sources. By situating badminton's development in Shanghai within its Republican-era social and institutional context, it reconstructs how the sport expanded from expatriate enclaves into local society. A decisive moment came with the Flying Shuttles, a university-based Chinese team whose victories in the late 1930s generated media recognition, institutional support, and the transformation of badminton into a mass urban sport. From the outset, women were visible participants, and badminton's distinctive format as a mixed sport made male–female co-presence in competition both thinkable and practicable. However, this integration was limited: while mixed doubles and co-educational classes symbolized modern equality, internal routines and public conventions reinforced auxiliary roles for women. In comparative perspective, Shanghai diverged from both British club traditions and settler-colonial adaptations by embedding badminton within civic reform and nationalist narratives, producing a paradoxical sporting space that appeared inclusive and progressive yet preserved subtle hierarchies. This study illuminates how modern Chinese sport became a site where gender, propriety, and modernity were constantly renegotiated.

## 1 Introduction

Badminton presents a distinctive case in the history of modern sport. Unlike many disciplines that only gradually incorporated women, badminton institutionalized mixed-gender participation at an early stage, with the Mixed doubles format first being included in major competitions as early as 1899 during the All England Open Badminton Championships (1). On the surface, this early co-presence of men and women has often been interpreted as a sign of relative progress, especially in contrast to the male dominance that characterized most modern sports (2). As a mixed-gender sport, badminton also spread globally along the routes of the British Empire (3), and in regions such as New Zealand, it was actively promoted as a light, mixed-gender pastime suitable for women (4, 5). While such promotion expanded opportunities for female participation, it simultaneously diminished the sport's perceived seriousness and reinforced its marginal status within the public and male gaze (4).

This apparent paradox raises a broader theoretical question: Does mixed-gender participation necessarily lead to gender equality? In contemporary sporting discourse, mixed-gender formats are often celebrated as symbols of progress, arenas in which men and women are assumed to compete on equal footing. Gender integration is thus associated with the potential to challenge forms of “gender injustice” in sport (6). However, the coexistence of men and women on the same playing field does not necessarily signal the dismantling of gender hierarchies; rather, it may conceal enduring forms of gender injustice beneath the surface of inclusion (7).

Such tensions must be understood within a broader rethinking of “gender” in the sociology and history of sport. Shifting understandings of “gender” have driven major paradigm changes in the sociology and history of sport (8), especially by challenging the fixed binary between male and female participation. For example, in the evolution of figure skating, “gracefulness” once symbolized aristocratic masculinity before becoming a defining trait of middle-class femininity, showing how shifting gender ideals are encoded in bodily expression and judged performance (9). In British lawn tennis, mixed doubles gained popularity as both a competitive and social format by the late nineteenth century. As Lake observes, while this format allowed women to appear in public competitions alongside men, it also subjected them to new norms of etiquette that reaffirmed their position as “protected” and secondary participants (10). Even in equestrian sports, often perceived as gender-neutral, women's inclusion unfolded gradually over several decades, with Olympic disciplines opening unevenly between 1952 and 1964 (11). These examples demonstrate that co-participation does not inherently lead to equality. Instead, it depends on how institutions script and structure gendered interactions. Against this backdrop, badminton offers an especially revealing site for examining the tension between symbolic integration and practical differentiation.

In contrast to its development in Europe, badminton's diffusion in Asia unfolded through a different trajectory. The spread of badminton in Asia was shaped by a more complex interplay of colonial legacies, state-building projects, community networks, and geography (12). Among Asian nations, China stands out most clearly. By the mid-1870s, badminton had already become popular among colonial elites in Singapore (12). By the early 20th century, the sport was well established in Indonesia (13). In contrast, badminton only began to appear in China in the 1920s. Despite being introduced to the sport relatively late, China rapidly developed into the most dominant force on the global stage, winning more World Championship gold medals than any other nation. The Badminton World Federation wrote on its official website, “China has been the most dominant nation, winning 70 gold medals. The second highest is Indonesia, with 23 gold medals, followed by Korea and Denmark” (14). This dominance was not confined to international competition, but also reflected badminton's domestic reach. General Administration of Sport of China's 2020 National Fitness Survey, badminton accounted for 8.9% of primary activities among adults aged 19–59, ranking behind brisk walking (22.7%) and running (19.8%) (15). China reported approximately 225,900 badminton courts nationwide, including about 193,200 outdoor courts and 32,700 indoor halls (16). These figures indicate widely accessible infrastructure and a broad participant base, suggesting that badminton functions as a routine urban pastime rather than a niche or elite pursuit. Compared with findings from New Zealand, where badminton was often portrayed as a light, feminized leisure activity and relegated to a secondary tier (4), China's mass uptake positioned the sport within both everyday recreation and competitive systems. Ubiquitous court access created conditions for men and women to play side by side, while state support for high-performance badminton offered parallel pathways for female athletes to gain visibility. These dynamics motivate closer examination of how China's trajectory challenged or reinterpreted gendered meanings inherited from badminton's Western origins.

Existing scholarship has established several important insights into the early development of badminton in China. Research has shown that badminton was introduced in the early twentieth century by Western expatriates and was first practiced through exclusive foreign clubs in major urban centers such as Beijing, Tianjin, and Shanghai (17, 18). Scholars note that Shanghai, a semi-colonial metropolis with overlapping jurisdictions, notably the International Settlement and the French Concession, offered a distinctive institutional setting for the sport's transmission (19). As one of the earliest hubs of modern sports in China, Shanghai also benefited from a dense media infrastructure and relatively well-preserved records, making it a particularly valuable site for reconstructing the early social context of badminton. Tian's research further demonstrates that by the modern period, Shanghai had already established a relatively mature club system and a broad grassroots foundation, laying the groundwork for the sport's continued development after the founding of the People's Republic of China (20). Evidence from the 1920s and 1930s suggests that local participation expanded during this period: while the Tianjin Young Men's Christian Association (YMCA)'s 1921 team was composed entirely of men, by 1932, female players had joined the ranks (17). The sport's growing appeal to women is also reflected in the example of actress Chen Yunshang, who reportedly trained at Saint John's University to improve her skills (21). After 1949, many Chinese returned from overseas and integrated the Southeast Asian badminton techniques they had learned with China's traditional footwork and strength, raising the nation's competitive level and driving the sport's post-introduction “re-Sinicization” (22). Furthermore, badminton has come to embody the national sentiment in China. Drawing on interviews and media texts, Tsang finds that Chinese badminton athletes often articulated their sporting identity through a discourse of “Chineseness,” aligning performance with collective, Sinicized ideals (23). In summary, the earliest forms of badminton in China were molded by expatriates and city clubs during the Republican period, and they persisted beyond 1949, becoming institutionalized and further re-Sinicized via returning overseas Chinese.

Despite these contributions, important gaps remain. Much of the existing scholarship has focused on tracing the introduction of badminton and outlining its institutional and infrastructural foundations. Yet it has not systematically explained the concrete mechanisms by which badminton spread beyond expatriate clubs into broader segments of semi-colonial Chinese society. Moreover, while individual cases of women's participation have been noted, the ways in which women entered the sport, the roles they assumed, and the discursive portrayals they received remain underexplored. Finally, little attention has been paid to how China's trajectory diverged from Western contexts, where badminton was often feminized and marginalized, and whether the Chinese case resisted or transformed these inherited gendered logics.

On this basis, this study undertakes a historical analysis of badminton's development in Shanghai during the Republican era (1912–1949), drawing primarily on contemporary primary sources. The present study poses three core questions: First, what institutional and social intermediaries facilitated the early spread of badminton in China, and what were the key moments and defining events in this process? Second, how did women enter and become represented within the sport, and in what ways did their roles and discursive portrayals change? Third, to what extent did the Chinese trajectory resist or transform the gendered meanings historically embedded in badminton, particularly in contrast to its Western contexts?

## 2 Method

This article is a historical study, grounding its analysis in primary sources from the Republican era (1912–1949). The principal data is drawn from the National Periodicals Index (Shanghai Library), a large-scale discovery system that provides access to tens of thousands of Chinese and foreign-language newspapers, with full-text coverage of the Late Qing and Republican eras. This resource preserves a wide array of rare materials and serves as a crucial gateway for reconstructing the early social history of sport in modern China.

**Figure 1:**
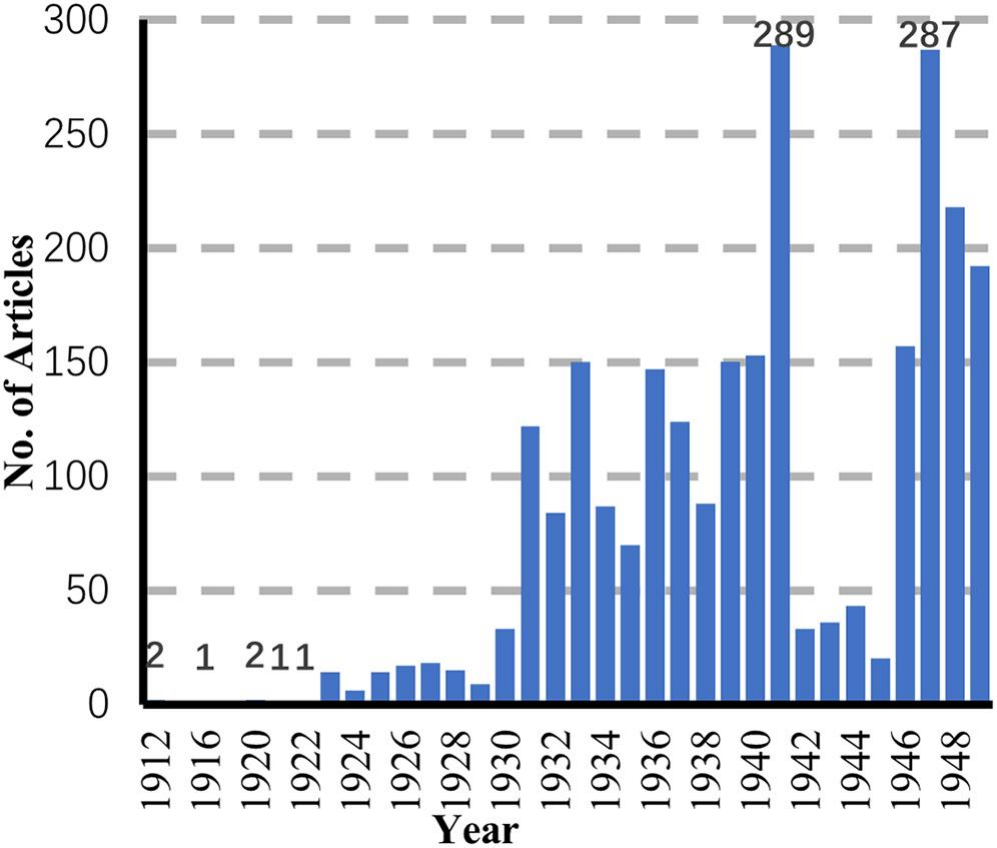
Badminton-related articles by years, 1912–1949. Bars show annual counts extracted from the index of national newspapers and journals.

Retrieval and selection. To build the dataset, this study conducted systematic searches using both Chinese and English keywords associated with the sport—“羽毛球,” “鸡毛球,” and “badminton”. From this process, as shown in Figure 1, this study assembled a corpus of 2,583 newspaper and periodical issues containing references to badminton during the Republican period. Each retrieved item was reviewed individually: trivial or passing mentions were discarded, while those containing substantive information about participation, venues, organizations, or gendered discourse were retained for analysis.

Analytical procedure. The retained texts were then contextualized within their broader historical milieu. Match reports, notices, fixtures, results tables, and feature essays were analyzed not only as factual records but also as cultural texts that reveal discursive constructions of gender and modernity.

Supplementary Materials to reconstruct regulations and competition formats, the press corpus was supplemented by contemporary badminton rulebooks and early editions of the Shanghai Sports Yearbook. These complementary sources provide critical detail on codified rules, organizational structures, and scheduling practices, allowing the study to situate media discourse within the broader institutionalization of the sport.

## 3 From winter diversion to rapid institutionalization 1912∼1934

### 3.1 The unexpected rise of badminton in Republican-Era Shanghai

After the opening of Shanghai's treaty ports, the city evolved into two principal foreign enclaves: the International Settlement, formed by the amalgamation of the former British and American concessions, and the French Concession, which remained under French administration (24). For the expatriate communities that occupied these districts, organized sport was understood as an integral part of daily life; they imported not only Western games themselves but also the attendant competition formats, governing bodies, and a range of purpose-built venues (25). Although The North-China Daily News—widely seen as the mouthpiece of the Shanghai Municipal Council—published an article on badminton as early as 1912, tracing its origins from India to Britain, this coverage was primarily derived from Reuters telegrams and targeted a foreign readership (26). It was not until 1921 that more systematic reporting on badminton reappeared in the press. Yet even then, the coverage still came from English-language newspapers rather than Chinese-language ones, indicating that badminton remained largely confined within expatriate circles rather than becoming a subject of broad interest in Chinese media or society.

During the First World War, Lady Annie de Sausmarez—wife of the senior British judge in Shanghai—founded the British Women's Work Association (B.W.A.) (27), to coordinate the sewing of bandages, garments, and other supplies for troops on the frontlines (28). In 1921, the association redirected that same female network toward leisure activities, creating a “Sports and Recreation Section” headquartered on the Bund. The new department organized monthly balls and afternoon teas and collaborated with the “Young Women's Christian Association” to stage hiking excursions, tennis outings, and badminton sessions for women (29). Badminton thus entered Shanghai's public sphere as little more than a seasonal curiosity, an indoor pastime for players who found tennis unworkable in the damp, chilly months.

The B.W.A., however, gave this “supplementary” sport an unexpectedly prestigious platform. Its inaugural event took the form of a full-scale tournament; the women of the B.W.A. who served in the interport badminton matches during the season will be specially invited to enter this tournament (29). Local journalists nonetheless expressed skepticism, dismissing badminton as a fad that “will soon be displaced by tennis” (30). In practice, early participants were indeed crossover athletes: tennis and cricket players who viewed a light racket and shuttlecock as a way to “keep fit in the winter” (31, 32). Consistent with that logic, the B.W.A. scheduled its badminton season to begin each year in October, directly after the close of the summer tennis calendar (33).

Yet demand quickly outstripped such provisional planning. The association's first sessions were held in the gymnasium of the “Public School for Girls” (34), but the facility quickly proved insufficient for the expanding membership. To relieve the pressure, committee members opened their private gardens on designated afternoons, though even they acknowledged that “this will scarcely support a large and growing club” (31). Within a year, the B.W.A. invested in a facility at its Bund headquarters and began inviting other organizations for friendly matches (35).

Limited courts were by no means the B.W.A.'s problem alone. The “Cercle Sportif Français” and the “Country Club” both erected temporary “mat sheds” in their gardens. The French club then went a step further, flooring over an unused swimming pool, adding electric lighting, and creating no fewer than a dozen regulation courts (36). As courts multiplied, inter-club fixtures became the next logical frontier. In Sportif Fran 1926, the French club publicly lamented that “a lack of inter-club matches is felt” and staged a series of exhibition games to attract wider interest (36). The Foreign Y.M.C.A. soon followed, highlighting mixed doubles so that “spectators among the fair sex might appreciate how strenuous the sport can be” (37). The All-England Open Badminton Championships included mixed doubles from its inaugural tournament in 1899 (1). So mixed doubles in Shanghai very likely emerged together with the introduction of badminton itself, since the format had already been firmly established in Britain by the late nineteenth century. Nevertheless, the earliest explicit evidence in the local press dates only from the mid-1920s. These spectacles effectively stitched together a rudimentary city-wide network.

Once matches spanned multiple venues and social circles, inconsistencies in rules, scheduling, and court dimensions became impossible to ignore. In response, the winter of 1928 saw the organization of Shanghai's first triangular series, contested by the Y.M.C.A, the Cercle Sportif Français, and the Country Club (37). A more durable solution followed in 1930 with the establishment of the Shanghai Badminton Association, led by President R. J. Martin and Secretary Mr. Stevens. Each participating club appointed one delegate to the central council (38). The association promptly launched parallel men's and women's leagues, each divided according to playing strength (38). Although mixed doubles were already popular in exhibition matches, this format was initially excluded from league competition. It was not until October 29, 1931, that the formation of a mixed doubles league was formally approved (39).

Contemporary reports emphasized the growing popularity of mixed participation and noted that, apart from the Portuguese Women's Association and the B.W.A., most women's organizations entered competition through their affiliated clubs (40). The Mixed League, organized into first and second divisions, was expected to become the most popular format (41). By 1934, the Shanghai Badminton Association had established a mature league structure with 29 teams (42). Mixed doubles was institutionalized as an official division alongside men's and women's singles, with entries from 9 clubs across two mixed-doubles divisions (42).

Within this system, participation was clustered in a clear club hierarchy. The Cercle Sportif Français was described as the radiating center of the game and operated seven high-standard indoor courts; the Country Club also ranked at the top, with its women's teams strongly favored in league and championship play (40). St. Joseph's Catholic Association and the Portuguese Company S.V.C. were identified as emerging contenders, while the Y.M.C.A., the Cathedral Badminton Club, and the Union Church Club remained second-tier but with growth potential (40). Against this backdrop, the B.W.A. appears as an exception: according to the 1934 league lists, it entered neither the women's bracket nor the mixed divisions (42). Contemporary reports cited the B.W.A. as evidence that women's badminton was “making progress.” Yet the association did not enter the league every year and thus rarely appeared in match reports or results. Its mode of operation also differed from most women's teams: the B.W.A. was not affiliated with the major mixed-gender club system and was typically noted only as an “exception.”

The rapid diffusion of badminton in 1920s Shanghai was closely linked to the active involvement of elite women's networks. Through social gatherings such as balls, teas, and philanthropic events, these women expanded the sport's visibility and lent it social legitimacy across both expatriate. As participation increased, various clubs responded by investing in purpose-built indoor venues, providing the material foundation for more regular and structured play. This institutional growth culminated in the founding of the Shanghai Badminton Association in 1930, which introduced standardized rules and organized citywide competitions. Mixed doubles also gained formal recognition during the process of institutionalization. Together, these developments enabled badminton to evolve from a seasonal leisure activity into an established component of Shanghai's urban sporting culture. But the B.W.A., an early driver of badminton's popularization, gradually faded from the league system.

### 3.2 Elite expatriate women fashioning badminton as a social statement

In 1920s Shanghai, the sport operated less as open competition and more as a coded marker of elite status, leisure, and gendered display. Contemporary English-language newspapers framed that dynamic with striking clarity. A 1912 feature in *The North-China Daily News* titled “History of Badminton, 'Hit and Scream'”—offered Shanghai readers a detailed genealogy of the game and emphasized its gendered trajectory in Britain:

“Badminton is one of the pastimes in which the standard of women's play is very high, yet strangely enough only two names were inscribed as lady champions for the first eleven years that it was played for.……till Miss Thomson had five and Miss Lucas six triumphs to her credit. Then the year came a change. Neither of the former lady champions was entered for competition, and new names soon filled the old and honourable ones. …… a prize better known in dowagering circles”(26).

The report thus underscored an apparent paradox: elite women could demonstrate remarkable skill, yet the championship roster remained practically locked to two recurrent winners because matches were confined to what the author called “dowagering circles”—that is, restricted groups of upper-class matrons whose numbers were small and whose primary interest was social interaction rather than athletic expansion. The passage set an interpretive template for understanding how badminton traveled to China: it portrayed embedded in class, gender, and social rituals rather than as a fully competitive sport.

In Shanghai, the most visible custodian of this transplanted culture was the B.W.A. From the outset, the association imposed a firm membership policy: “only British women who are members of the British Women's Recreation Section are to be allowed to play on the courts … women of any other nationality may be allowed to play as guests, but in limited numbers” (29). This by-law demonstrates how badminton reinforced both imperial identity and gender exclusivity; it fenced off the courts as a space for British femininity while relegating other nationalities to the peripheral role of occasional invitees. Internally, the B.W.A. favored sociable formats over strict ranking tournaments, notably the American draw-system competitions in which doubles partners were assigned by lot to maximize mingling rather than to crown definitive champions (43). Yet the association did not remain entirely insular. Contemporary newspaper articles reported that the Sports Section had “run a successful badminton club for several seasons” and that “the Sports Section will be pleased to hear from any clubs wishing to arrange ladies’ matches” (44). In practice, the B.W.A. organized friendly fixtures with the Country Club (35) and the Portuguese Women's Association (45), thereby positioning itself as a gatekeeper and broker in an emerging inter-club network spanning women's and mixed-gender clubs. So outward engagement bolstered the association's prestige while leaving its internal hierarchies intact.

Because competitive depth inside single women's clubs was limited, exhibition play—especially mixed doubles—became the preferred vehicle for publicity and recruitment. At a Y.M.C.A. Open House event, for example, organizers highlighted that “there will be mixed doubles where Mrs. Burton and Miss Seaborne have consented to play … there will be a good opportunity for members and guests to witness the matches in between the dancing which more or less features the occasion” (37). Here badminton was slotted between ballroom interludes; the spectacle rested on two female players whose presence promised to “brighten” the card and lure additional women spectators. A similar formula guided the Cercle Sportif Français in 1926. Arguing that inter-club encounters were still too sparse, the French organizers staged demonstration matches and advertised the sport's benefits in expressly gendered terms: “if more of the fair sex knew what wonderful exercise can be got from a light racket and shuttle, there would be many more playing” (36). Women's appearances were curated as conspicuous moments of display rather than routine elements of a formal schedule; they functioned as living posters for a sport officials hoped to broaden within acceptable social parameters.

Period reportage also dwelled on the theatrical dimension of female participation, detailing sartorial choices alongside technical prowess. One column praised women as the best net players—“The best net-clears are women also … civility is only held like armour under a sheepskin grin” (46)—while others focused on sartorial choices: “Wearing fur coats; colorful sports jackets … gold bangles above the elbow … short hairstyles such as shingled, bobbed, or Eton-cropped, with optional Marcel waves” (47). Observers noted how a player might nonchalantly toss her fur coat onto a sideline chair, retain her vibrant sports jacket for the opening rallies, and only then strip down to a sleeveless dress as a psychological ploy against an opponent. Such descriptions reveal badminton less as a purely athletic contest than as an enacted tableau of fashion, grace, and polite aggression.

These materials point to a through-line: in 1920s Shanghai, badminton was cultivated as a social asset for elite foreign women. This social orientation became one contributing factor in the B.W.A.'s later fade from the league. Regulated by nationality and guest rules, scheduled around teas, dances, and garden parties, and showcased through exhibition mixed doubles, the association accrued visibility without building the competitive capacity required for league entry. Without stable teams and routine fixtures, the B.W.A. did not translate early prominence into league participation, and its presence diminished over time.

## 4 From champions to citywide craze (1935–1949)

### 4.1 The breakthrough of a Chinese student association in Shanghai's expatriate-dominated badminton field

In the mid-1930s, “Shanghai already sustained a broad ecology of badminton with events at various types and levels” (40). Nevertheless, participation remained socially narrow, embedded in Western club culture, and the sport was widely viewed as an aristocratic pastime with limited uptake (48). The tennis players Khoo Hooi Hye (邱飞海) and Lum Pao Hua (林宝华) were among China's earliest celebrated figures in the sport. In 1927 they partnered to win the tennis championship at the Eighth Far Eastern Championship Games held in Shanghai (49). Both athletes likewise promoted badminton, portraying it as an appropriate indoor sport for the winter season and, once the tennis season had ended, as the only effective exercise for the arm (50). Khoo emphasized that badminton was “suitable for both men and women and full of interest, and it was hoped that the nation would rise to practice it” (50). In the early 1930s, the tennis player Cannavaro promoted badminton, converting one of his tennis courts for badminton use and offering instruction in Shanghai (51, 52). One commentator further argued that the game deserved active promotion, yet it failed to achieve broad popularity at the time (53).

Although badminton had not yet achieved wide popularization, early Chinese promoters helped bring the sport onto school campuses. In 1935, *Minbao* reported on the Shanghai Municipal Girls' School summer sports day, which showcased the modern female body and included multiple ball games, basketball, volleyball, badminton, tetherball, and softball held concurrently, with invited girls' schools participating (54). Meanwhile, badminton also spread through boys' schools: in 1935, it was introduced at the S.M.C. Public School for Chinese (39), and in 1936, the school staged singles and doubles tournaments for students and teachers, widely noted as the first reported on-campus badminton event in Chinese schools (55). At St. John's University, initially not co-educational, co-education expanded by 1936 (56). In the same year, students independently founded the Flying Shuttles and launched a campus Singles Handicap Tournament, awarding a silver cup to the winner (57). These developments did not occur in a vacuum: the organizational capacity of student groups and rising visibility in the Chinese-language press together enabled a shift from informal play to formal competition. While women were visible in school sports days, the formalized campus badminton tournaments publicized at this stage were predominantly male.

In 1936, the Flying Shuttles, competing under the name St. John's University, entered the Second Division of the men's league (often styled “junior division,” a label denoting standard of play rather than age). They were the only Chinese team that season and captured the division title in their debut campaign (58, 59). Team members also contested the season's individual championships and represented China in the international “Whalley Shield” (60, 61). Chinese-language newspapers portrayed these results as breaking an exclusively Western preserve (62). English-language newspapers reframed this success as a broader inflection point for Chinese participation. *The Shanghai Evening Post and Mercury* declared:

…all that badminton needs to make itself really popular in Shanghai is for St. John's to end up at the top of the junior division this year and go on to win the senior title next year or the following annum. Local Chinese would take up the sport, and with the natural aptitude and some specialized and scientific training and coaching, such as the China National Amateur Athletic Federation may be ready to provide, the Chinese should adopt badminton as one of their national games (63).

Citing results “to date without a single defeat being chalked up against them,” this news judged that “for a team that is entered in the league for the first time, this record is no mean achievement” (63). The press bound a student team's performance to a narrative of Chinese uptake and nationalization of badminton, thereby challenging the symbolic monopoly long held by Western clubs.

War temporarily broke this momentum. With the 1937 Battle of Shanghai and the Japanese occupation of St. John's campus (64), “from the autumn of 1937 to the summer of 1938 the association discontinued its activities, as most of its members left the college because of the hostilities in Shanghai” (57). Although core players returned in autumn 1938, postal delays prevented league registration; nevertheless, St. John's University's Tonnie Wang and Timmie Tseng represented China at the 1939 Whalley Shield (57). Indicates that the war did not keep this student association dormant for long.

After women were admitted, the Flying Shuttles' mixed doubles squad achieved notable success in the 1940–1941 season: “their newly formed mixed doubles team also won the mixed group championship” (65). By 1940, the group reappeared not as a university club but explicitly as the Flying Shuttles, signaling a shift from campus affiliation to a semi-autonomous Chinese athletic organization. That season, they “crowned their season's efforts in the Badminton League” by defeating C.S.F. “B” 9–0 at the French Club, building “a record which will be impossible for the other teams in the competition to duplicate” (66). The China Press then offered extended coverage of the club's origins and development (57). The renaming and press legitimation marked structural consolidation: Chinese participation had moved beyond symbolic entry to an enduring club identity within the league's structure.

In 1941 this consolidation found its personification in team captain William Funk (洪德全), whose dominance—men's singles, men's doubles, and international doubles—was headlined as “Ace Badminton Player Wins Three Honors” (67). As The North-China Daily News put it, “much of the credit of the success of the *Flying Shuttles* must also be attributed to Funk as it was his presence in the No. 1 position that permitted his team to score sweeping victories in league play,” and “none other than William Funk of St. John's University—has walked off with practically every badminton honour that this city has to offer” (68). A Singapore-born Chinese who “picked up the game in 1935,” Funk won the Junior Singles Championship of Singapore in 1937, and became the undisputed champion of St. Joseph's College after moving to Shanghai (68). Funk also authored *Good Badminton*, which was scheduled for publication in mid-October 1941, hoping “to raise the general standard of badminton in Shanghai… [and] giving lectures to local badminton enthusiasts” (68). His trajectory marks a shift from mere competitive success to localized knowledge production and instruction, strengthening Chinese authority within the sport's technical discourse.

As badminton moved into Chinese social worlds, it likely first found a foothold in Western-style mission schools. The Flying Shuttles exemplify how a student association led by overseas Chinese converted school-based resources and early league success, navigated wartime interruption, re-emerged under a club designation, and culminated in star-centered knowledge production. This progression demonstrates not merely a symbolic breakthrough but a measure of structural incorporation of Chinese actors into Shanghai's expatriate-dominated badminton field. Because St. John's was among the earlier universities to expand co-education, it could field mixed-doubles teams, indicating that at this stage, mixed doubles were likely concentrated in co-educational institutions.

### 4.2 Champion momentum and the badminton boom

With badminton's entry into Chinese social life, certain young men dismissed it as “women's play,” arguing that the sport's “light racket, light shuttle, and small court” were “insufficiently vigorous and powerful,” and therefore “unsuitable for young men” (69).

This gendered imagination was reflected in the visible user base at the outset, which consisted largely of young women from the upper strata. One account recorded that “recently schoolgirls have suddenly taken to badminton after class … besides their textbooks they all carry rackets in their hands, even bringing them into the classroom” (70). Observing the scene near a school, the writer Liu Xu confessed, “I was much taken with it,” yet “ashamed that my youthful years were passing,” and thus “did not feel it proper to join.” The vignette ends in sensory detail: rackets rising and falling, sunlight glinting on “the girls’ fair wrists,” the sound of strokes mingled with laughter, all “irresistibly alluring” (70). This rhetoric foregrounded appearance rather than exertion, and it marked age and gender as tacit boundaries to participation. The players often hailed from institutions like St. Mary's, the McTyeire School, and St. John's University, schools considered “somewhat Westernized” where playing an imported sport was said to be “only natural” (71). St. John's in particular had “high tuition and the reputation of an aristocratic school” (72). Before badminton achieved citywide popularity, it was concentrated among women in Western-style elite education. This early configuration closely resembled the pre-league scene of the 1920s, centered on upper-class women's recreational networks such as the B.W.A. The sport functioned more as fashionable sociability than as a competitive pursuit (73). As the league structure took shape, organizations like the BWA gradually lost prominence within the new competitive framework, while in the late 1930s the rise of the Flying Shuttles team helped unsettle the prevailing notion that badminton was primarily a women's pastime.

In 1936, *the Shanghai Evening Post and Mercury* observed that if St. John's University ever claimed the league title, it would spark a true badminton craze in Shanghai (63). At that time, Chinese-language newspapers had shown little interest in the sport, which remained marginal in local Chinese society. However, the Flying Shuttles' dramatic rise—securing the league's top-tier championship in 1940, successfully defending the title in 1941, and winning an international tournament under the banner of “China”—triggered a wave of media enthusiasm. *Shun Pao* began regular coverage of the *Flying Shuttles*’ matches across local competitions, and smaller papers quickly followed suit. *The National Herald*, in particular, showered the players with praise, referring to the St. John's athletes as “Chinese champions” (74), declaring Funk “Shanghai's overall champion” after his singles victory (75), and lauding their unprecedented achievements as “mission accomplished” (76). These accolades transformed their victories into public milestones; the Flying Shuttles' success no longer belonged only to their university or a club catalyzing badminton's transition from elite pastime to a sport with broader societal appeal —it resonated with a broader national imaginary.

This sporting success produced swift ripple effects in Chinese civil society. In 1942, the Dahua Badminton Club became one of the earliest documented Chinese badminton clubs to recruit members through Chinese-language media (77). Commercial gymnasiums added courts (78), and elite players such as Funk were invited to exhibition matches across the city (79). Even the film star Chen Yunshang was known to play badminton at St. John's University and was publicly associated with the sport (80). Badminton thus gained a new identity: fashionable and aspirational, associated with youth, modernity, and national pride.

The wartime regime change in 1943 provided structural opportunities for institutional reform. As the Wang Jingwei-led Reorganized National Government took control of Shanghai (81), new spaces emerged for Chinese actors to participate in governing the city's cultural life, including sport. The Overseas Chinese Sports Association seized this opportunity to organize the “Huaqiao Cup,” a badminton tournament explicitly aimed at Chinese athletes; the Flying Shuttles were among the teams that entered (82). The association further promoted badminton by inviting renowned male and female players, both Chinese and Western, to participate in exhibition matches—once again featuring members of the Flying Shuttles such as Funk (83). Other civic institutions, such as the YMCA, also invited the team for performances (84), solidifying their celebrity status in the local athletic scene. In the same period, municipal authorities restructured badminton governance. The Shanghai Badminton Federation was established under the Special Municipal Sports Association, with a board that included both Chinese and Western figures (85). Flying Shuttles members Fred Li and S. C. Hou served as honorary treasurer and officer, respectively (86). In addition to *the Flying Shuttles*, newly formed Chinese teams such as the “Huaqiao Association” and “Yucai” joined the league and competed alongside expatriate clubs (87), marking a major shift: Collaboration between Chinese and Western actors increased, and Chinese voices gained unprecedented institutional weight in shaping the direction of the sport.

By 1944, this convergence of sporting success, media amplification, and institutional transformation had produced a full-blown badminton boom. Courts proliferated not just in clubs and gyms, but in parks, alleyways, and even on city streets (88, 89). Reports noted that “in Shanghai, badminton has had a long history … but it was not until this year (1944) that it became popular, which leaves one puzzled as to the reason” (90). However, the Flying Shuttles' championship victory and the public recognition of its members offered a new image of badminton—one that demonstrated strength, skill, and competitiveness. This high-profile success contributed to altering public attitudes and helped legitimize badminton as a modern sport appropriate for both men and women. Meanwhile, the press actively countered charges that badminton was “too feminine,” seeking to recast its bodily feel and athleticism. “Many people told us that badminton is a sissy game, but a recent visit to one of the local courts convinced us that they were talking through their hats” (91). “Strong men have crawled off the court on their hands and knees after a hard game” (91). Chinese-language reports likewise stressed full-body training: “With prolonged exercise, the muscles of the whole body gradually become firm, fat gives way to muscular beauty” (92). They also underscored that the belief that badminton was merely “women's play” stemmed from ignorance of its intensity: “one should not judge rashly without firsthand experience” (69). By around 1944, at the height of the boom, the sport's gendered meaning had been redefined from a graceful diversion for women to a modern activity suitable for both sexes and capable of high intensity. Contemporary news remarked: “Originally badminton was a sport played specifically by women. Now there seems to be gender equality, therefore it is no longer divided by sex” (90).

The sport's popularity provoked municipal interventions: park authorities banned play on the grounds that it damaged plants and obstructed other visitors (93), while traffic accidents and injuries caused by street games led to further police restrictions (94, 95). Yet these interventions only confirmed badminton's reach. In 1944, the news described young men and women with rackets everywhere (90). It had become, in the words of contemporary observers, “the most fashionable pastime” in Shanghai (90).

### 4.3 The expansion of badminton and the gendered friction of mixing

Following Japan's surrender in 1945, the Nationalist government took over from the Wang regime (96), and the sport's administrative order was again restructured. The Shanghai Badminton Federation was replaced by the Shanghai Badminton Committee under the Athletic Federation (97), and league competition resumed (98). Yet despite the political transition, the Flying Shuttles continued to dominate. *The China Press* declared: “There is one team which is destined to make a name for itself. The team concerned is the Flying Shuttles” (99). During a commemorative exhibition aimed at “arousing public interest in the game and securing support of all clubs” honoring Sun Yat-sen's birthday, over 1,000 people watched the team's members, including Funk, perform in a match (100). “The spectators went away agreeing that a more fitting observance of Dr. Sun Yat-sen's anniversary could not be found” (101). This ritualized visibility not only affirmed the team's popularity but also helped the new committee attract a broader audience.

In the mid-to-late-1940s, participation expanded rapidly. In 1946, 16 teams entered the league. But in 1947, 46 teams participated (102, 103). In 1946, the YMCA promoted mixed-gender exercise classes on a regular basis, with badminton among the offerings (104). In Tianjin, the YMCA even assigned dedicated instructors and charged no fees (105). The Shanghai Badminton Committee followed suit, designating three matches in the mixed B division as free-admission events to encourage spectatorship (106). These efforts helped establish co-presence as the norm in badminton's popularization. Competitive routines also became more integrated. Public tournaments increasingly featured mixed-gender and mixed-heritage pairings: in 1947, the Flying Shuttles' member Kay Wong partnered Jiggic Rozario to reach the women's doubles final (107); in 1948, Funk won a second open title of the season by partnering Mrs. A. B. Wilkinson to capture the mixed doubles championship (108). Such pairings normalized cross-community play and weakened older club segregations, embedding inclusivity within elite competition.

At the same time, public discourse surrounding the sport underwent a significant transformation. In 1934, Chinese-language commentary still described badminton as a “noble” indoor pursuit (48, 109). By the late 1940s, newspapers emphasized their affordability and suitability for children, women, and the elderly, presenting them as “not too intense” yet beneficial for health and discipline (90, 110–113). This shift did more than broaden the sport's social appeal; it redefined the meanings attached to participation. In the context of Chinese modernity and urban governance, such representations aligned closely with contemporary efforts to regulate gendered leisure. Civic advocates in the Republican era promoted “proper entertainment for women,” exemplified by Mrs. Chen Yongsheng's Saturday afternoon badminton classes (114). More importantly, badminton became incorporated into projects of urban governance and moral regulation. During the implementation of the dance ban, one news outlet proposed a reallocation of social space: large ballrooms could be converted into basketball courts, smaller halls into tennis or badminton venues, and the smallest rooms into tables for table tennis. They added that “using female partners for play should not fall under the prohibition on dancing” (115). In this context, badminton was reimagined as a gender-paired alternative to ballroom dancing, offering a “proper” form of interaction between men and women while maintaining moral and social boundaries. These developments indicate that badminton in Shanghai was not merely adopted from abroad, but actively reconfigured within local social, cultural, and political contexts. The transformation of discourse thus did not merely reflect changing attitudes but actively legitimized mixed-gender play by framing it as accessible, moderate, and civically beneficial, thereby reducing the perceived risks of cross-gender interaction.

These shifts also reinforced ongoing institutional initiatives, including league expansion, Y.M.C.A. programs, and free public matches, which together normalized mixed-gender participation. Robertson argues that globalization should be understood as a process of “glocalization,” in which global and local forces interact simultaneously rather than in a unilinear manner (116). The Shanghai case demonstrates how badminton, as a global sport, was transformed into a culturally specific and socially regulated medium of gender interaction through this process.

Nonetheless, the move toward inclusion did not eliminate gendered inequalities. On the contrary, new tensions surfaced in practice. One account noted that when a woman played with notable force, “onlookers brazenly mocked and harassed her,” behavior described as typical of “lecherous men” (117). Authorities grew concerned that badminton, once considered suitable for girls, had become disruptive when played by rowdy young men, reportedly “spoiling the park's atmosphere and blocking visitors’ paths” (118). More disturbingly, incidents of gender-based violence were reported. In 1948 one news recounted that in Zhongshan Park, “a fifteen-year-old girl, Yang Gendi, playing badminton with her younger brother, chased a shuttle blown by the wind; as she stooped to pick it up, a plainclothes soldier pinned it with his foot and took the opportunity to molest her … when the girl angrily rebuked him, the soldier fired a shot at her”, after being rushed to hospital, she remains in critical condition (119). Another news report mentions that, “Student Yao Huizhong went to Zhongshan Park with his girlfriend Lu Xiuqing and another girl surnamed Ma. While they were playing badminton, three men in suits harassed Lu and Ma, which led to an argument. As they left, the men returned with another in uniform; one claimed ties to the gendarmerie and threatened them. The uniformed man then drew a pistol and fired at Yao, missing him but injuring a bystander” (120). In the wake of these conflicts, women's visibility within public badminton scenes declined. A retrospective from 1949 noted: “At the beginning of its vogue, eight years ago … before Fuxing Park, there were many schoolgirls batting the shuttle every day … Badminton remains the same, but it has now been abandoned by us schoolgirls. Today, it is the boys in primary and middle schools who are all playing it” (121). This indicates that even as mixing became a consensus in institutions and discourse, women's athletic space in everyday practice was compressed. Gendered frictions in public space pushed women to withdraw from settings they once led, altering the sport's gender composition.

To summarize, the late 1940s saw badminton move toward a more inclusive and mixed format, supported by institutional expansion and changing media discourse. However, this shift remained uneven. While formal participation broadened, gendered tensions in public spaces constrained women's visibility, revealing the persistence of social boundaries beneath surface-level inclusion.

## 5 Negotiating gender through mixed sports in Republican China

### 5.1 Men and women: a bifurcated badminton culture

Contemporary reportage asserted that “there is no par in badminton. Anybody can play it. It is an ideal mixed game, women playing the net and men the backcourt” (122). This inclusive formula invited readers to imagine badminton as open to all and to view mixed participation as both ordinary and desirable. Yet the very discourse that heralded inclusivity also generated a second interpretive track directed specifically at women. Under the premise that “women should do exercises suited to themselves because their muscular strength and explosive power differ,” badminton was recommended as a gentle, healthful activity appropriate for women (123). Medicalized language gave this premise a tone of expert authority. Commentators claimed that “the muscles of the chest, through respiratory action, gradually develop,” and that the sport was “of particular benefit to women,” especially in a society where “most women in our country have underdeveloped chests and insufficient lung capacity” (92). Others added that “many women in our country suffer from stomach ailments,” hence “badminton is urgently recommended” (124). Practical advice columns reinforced the same message. The “Women and Family” page in *Shun Pao* urged readers to “choose ball games that are not strenuous,” concluding that “badminton is quite suitable” (112). Together, these strands of rhetoric positioned badminton as simultaneously universal and feminine. The result was an abiding tension between the public claim that “anybody can play” and the promotional claim that women should play because badminton is non-strenuous, corrective, and safe.

Institutional rule-making carried this discursive bifurcation into the codified structure of competition. The 1940 Shanghai Sports Yearbook introduced badminton as a sport “people of all ages and both sexes can give a try,” while immediately specifying a sex-based scoring differential: “matches may be played to 15 or 21 points per game; women's singles are played to 11 points (although a 15-point game is also acceptable)” (125). In principle, that “note” preserved a space in which women might be scored like men. In practice, subsequent publications narrowed that space. The 1943 Modern Sports handbook stated that “men usually play to 15 or 20, women to 11” (126). In 1946, the rules approved by the Shanghai Special Municipal Sports Association repeated the pattern: “matches may be played to 15 or 21” and “women's singles to 11” (127). By 1948 an officially endorsed ruleset published by Zhongzheng Book Company went further, prescribing best of five for men and best of three for women, while retaining 11 points for women's singles (128). Read as a sequence, these texts trace a movement from ambiguity to clarity. What began as a permissive note that allowed women to play to 15 points hardened into a standardized separation in both points and match length. The differentiation was not only a local custom. A 1949 London manual, An Introduction to Badminton, summarized international practice in identical terms: “The doubles or men's singles game consists of 15 points (or 21, if this is arranged beforehand). Women's singles usually go to 11 points” (129).

It should be emphasized that gender distinctions in badminton during this period were manifested not only in the rules governing games and scoring, but also in players' positioning on the court and the allocation of roles. For example, the “Up and down’ formation… (this) formation is particularly suited to mixed doubles” (129). This division of roles was not seen only in the mid-1940s. Tactical literature on mixed doubles extended this formalization from rules to roles. The 1939 New York volume Better Badminton instructed that “The up (net) player should only take overheads she can place safely… Her main aim is to steer rallies so opponents lift to her partner; she should never clear to the opponents’ stronger partner. She must not back into her partner's space, maintaining clear up-and-back roles” (130). Although presented as neutral advice, the language assigns a script to each body. The assumed “she” occupies the front court as controller, directing tempo through drops, blocks, and interceptions. The assumed “he” occupies the back court as finisher, empowered to attack with full overhead strokes. The instruction is not a statistical description of outcomes. It is a prescriptive blueprint that normalizes a division of labor and wraps it in the rhetoric of safety, efficiency, and good partnership. Contemporary Shanghai reportage affirmed these allocations through praise and blame. A 1941 article in The North-China Daily News chronicled a mixed-doubles match between the Flying Shuttles and the French Club. It praised “Winnie Cheung,” who “camped at the net, pressed mercilessly whenever a return shot could be intercepted, and she never relented in going on to win true championship style.” By contrast, “Mrs. Jorgensen” was criticized for attempting to smash from the back court, an act the writer treated as ill-judged and even decisive in the loss (131). The author was Flying Shuttles captain William Funk, a figure of technical authority. He explicitly affirmed the “women in front, men in back” configuration and construed departures from it as evidence of failure.

Taken together, these developments reveal a clear cultural bifurcation in the course of badminton's popularization. On the one hand, public discourse celebrated it as an “ideal mixed game,” ostensibly open to all. On the other hand, women were medicalized as suited to “gentle exercise,” assigned different point values and match formats by the rules, and fixed in auxiliary forecourt roles within mixed-doubles tactics. In concrete match reports, female players were even faulted for deviating from this division of labor. “Mixed,” in short, did not signify equality in either principle or practice; it operated through gender partitions and role differentiation.

### 5.2 Interpreting different sports through the lens of the “new woman”

In Kramer's analysis, a nationalist narrative refers to the ways in which nationalism is explained by defining who belongs to the nation, distinguishing insiders from outsiders, and establishing norms about proper behavior and identity (132). Such narratives establish boundaries between “proper” and “improper” practices and provide justification for particular forms of social organization.

These narratives were historically embedded in the ideological context of Republican China, particularly in the discourse of the “New Woman (新妇女)” and the National Salvation Movement. Following the May Fourth era, women's bodies and social roles were redefined in relation to the national crisis and modernization. Female participation in sport was framed not only as a matter of personal health, but as a contribution to national strength. Public discourse oscillated between the rhetoric of “physical culture for national strengthening” and that of “healthy beauty”, while the figure of the female athlete emerged as a symbol of a modern female elite (133).

At the same time, sport became increasingly linked to national and ethnic identity. In 1949, when a Malaysian-Chinese team won the inaugural Thomas Cup, Chinese newspapers hailed the victory as “a glorious chapter in Chinese sports history” (134). The *Shun Pao* emphasized ethnic identity over nationality, describing the victory as “The Chinese did not invent Badminton, but the first Thomas Cup belongs to Chinese hands… proof of a natural talent for the game of Chinese” (135). Despite the fact that the players did not hold Chinese nationality, the media celebrated their ethnic identity, echoing the earlier praise accorded to the Flying Shuttles and their captain, Funk, a Chinese from Singapore who had risen to prominence in Shanghai's badminton scene. These reactions suggest how badminton, by the late 1940s, had become deeply embedded in ethnic nationalist sentiment. The sport was no longer seen only as a colonial pastime. It had gradually become a space where Chinese people could participate and be recognized.

More importantly, these discourses shaped a specific cultural psychology and social perception through which gender and sport were understood. As scholars have noted, “In modern China, the female body has been imbued with multiple symbolic meanings, representing dichotomies such as ‘East–West’ and ‘tradition–modernity’. The involvement of women in sports has been seen as a transitional phase in social modernization, influencing the self-perception and body image of the ‘new woman’” (136). Within this framework, women's physical practices were not viewed as purely individual activities, but as symbolic expressions of broader cultural and national transformations. Participation in sport thus became a site where expectations, traditions, and priorities were negotiated. These tensions were further reflected in contemporary debates over women's sport. The question of whether women's sports should adopt sex separation remained unresolved in Republican-era China. Sources reveal three main positions: first, that most sports played by men should also be open to women; second, that high-intensity training should be limited to avoid injury; and third, that arrangements should be made case by case rather than based on fixed rules (133). These debates shaped not only national policy but also local implementation in urban sport scenes such as Shanghai. Many promoters of women's sport in the Republican period came from missionary school backgrounds and had studied abroad. They drew on Western physiological knowledge and advanced a program of gender-neutral rules in order to raise women's competitive standards (136). However, such efforts often encountered public resistance. For example, the educator Lu Lihua, a pioneer of women's physical education, once organized a basketball match between a women's team and a men's team from the Liangjiang Women's College. The event was heavily criticized in the press as “inappropriate” and “improper” (133), revealing persistent anxieties about women crossing gendered boundaries in public sport.

Badminton provides a concrete site where these perceptions were translated into practice. Introduced from Western clubs, badminton in China did not undergo an extended process of cultural indigenization. Its competition rules and its tacit gender logics were transferred largely intact. The notion of an “ideal mixed game” circulated widely in both publicity and reportage: “…there is no par in badminton. Anybody can play it. It is an ideal mixed game, women playing the net and men the backcourt” (122). This phrasing is deliberately universal, inviting readers to imagine co-presence as the normal condition of play and to accept mixed participation as modern and respectable. Yet the egalitarian tone of that formulation quickly met the friction of practice. In mixed doubles, the default pattern of “she in the forecourt, he in the backcourt” did not arise by chance at courtside. It reflected an institutionalized settlement that justified role separation in the name of physiological difference and safety. Rather than loosening gender boundaries, the mixed format often confirmed and rationalized them as technique. This positioning was not purely technical, but was made meaningful through prevailing social perceptions of gender. In a context that emphasized collective strength and social order, clearly differentiated roles could be understood as functional and appropriate, and thus came to appear as a natural and efficient organization of play. The cross-sport career of Kay Wong (黄美娟) shows how this tension was lived and narrated. Kay joined the Flying Shuttles in 1941 (137) and graduated from the English Department of St. John's University in 1943 (64). In her early seasons, she was not a core player and had limited access to marquee fixtures. Following the team's reorganization as Chung Hwa and the Flying Shuttles Reds in 1946 (138), she became one of the team's mainstays. As a top Shanghai player, she was invited to exhibition matches organized by the Shanghai Badminton Committee, with mixed doubles as her primary showcase (139). Yet even in these appearances, contemporary accounts often centered on her male partner. For instance, a newspaper described one match: “with the set point within the losers’ grasp, K. W. Choy made a brilliant recovery and partnered Miss Wong to tie the count at 14–14” (140). This phrase foregrounds Choy's action while relegating Wong to a supporting role. During the St. John's period, the strongest women's results in the club's record were similarly concentrated in mixed doubles rather than in the women's draws. Only in the Chung Hwa Flying Shuttles period did Kay begin to appear regularly in women's singles and women's doubles. There, she quickly distinguished herself: she was the only Chinese entrant among six players in the women's singles draw (141) and was listed as “top seeded in the women's singles,” a credible contender for the title (107).

Kay's basketball career clarifies the contrast. She served as forward and captain of the women's basketball team of the Shanghai Dahua Sports Association (142). After a loss to a strong opponent, she gave a measured assessment: “The failure was anticipated and therefore not a matter of concern.” (143) Newspapers described her as “long-experienced and especially skilled, of commanding caliber” (144). The team's request to reschedule a final because her injury had not yet fully healed indicates the degree to which the team's organization and tactics were built around her presence (145). Compared with mixed doubles in badminton, the women's basketball context afforded a woman clearer authority in both strategy and leadership. Within the single-sex format of women's basketball, her role encompassed tactical authority and symbolic leadership, roles not equally accessible in the mixed-gender context of badminton.

Against this backdrop, Kay Wong's experience in both basketball and badminton offers a valuable point of comparison. This contrast highlights how different institutional settings shaped gendered participation. While women's basketball provided a space for female authority, badminton, especially in mixed doubles, channeled women into predefined roles. Kay Wong's experience thus demonstrates how gendered meanings in sport were shaped not only by institutional arrangements, but also by how participation was structured and understood. More specifically, her contrasting roles in basketball and badminton reflect a broader tension in Republican China, in which women's participation in sport was encouraged as a sign of modernity, yet simultaneously constrained by expectations about appropriate gender roles.

## 6 Conclusion

This study set out to explain how badminton moved from expatriate clubs into local society in semi-colonial China, how women entered and were represented within that process, and to what extent the Chinese trajectory reworked the sport's inherited gender meanings. The evidence shows that popularization was not a single leap but a sequence in which social networks, institutions, and media repeatedly lowered entry costs and stabilized new routines.

First, the evidence shows that the popularization of badminton was not a top-down initiative, but rather a layered process driven by social networks, institutional actors, and public media. In the 1920s and 1930s, elite clubwomen and missionary school alumni helped introduce the sport into local educational and recreational spaces. Venues such as the YMCA, school gymnasiums, and park courts provided the material basis for expansion. A pivotal moment came with the formation of the Flying Shuttles, a team led by overseas Chinese students. Their consistent victories in the mid- to late 1930s generated widespread media attention, encouraged the emergence of new Chinese badminton clubs, and facilitated institutional inclusion amid wartime administrative reorganization. As a result, badminton evolved from a seasonal pastime within expatriate enclaves into a mass sport integrated into Shanghai's urban life.

Second, women did not enter as peripheral figures. Early uptake clustered among students in Western-style schools, which made badminton visible as a fashionable and female-friendly practice. Mid-1940s institutions then normalized mixed participation through classes and league formats, while press rhetoric recast the game as modern, healthful, and suitable for both sexes. At the same time, the sport's internal scripts and public settings placed limits on women's roles. Mixed doubles routines positioned women at the net and men in the back court; scoring and match-length conventions marked out sex-differentiated singles; and frictions in parks, including harassment and isolated violence, compressed women's everyday visibility. Women-only brackets, by contrast, offered a wider field for individual selection and recognition.

Third, in comparative perspective, the Shanghai path neither repeated New Zealand's relegation of badminton to a gentle secondary tier nor simply reproduced British club etiquette. Ngo and Watson's study of New Zealand shows that in the 1920s to 1930s, badminton was persistently labeled a “winter supplement.” The press framed it in the idiom of “indoor lawn tennis,” which suppressed its independent identity and promoted it as a gentle pastime “suitable for ladies.” (4) This raised women's participation but at the same time reduced the likelihood that men and the wider public would regard it as a serious competitive sport (4). Shanghai followed a similar early path, but its later evolution was shaped by unique conditions. Badminton formats in Shanghai were institutionalized at the league level and attached to ethnic and national narration that made Chinese participation legible and valued. Although badminton was introduced to China during its semi-colonial period, Chinese society did not simply replicate British club culture. While the core rules and etiquette were preserved, the sport developed distinctive local forms. It was played primarily in schools and public gymnasiums, which were semi-open venues rather than exclusive private clubs. Its dissemination was led by returnee students, educators, and local youth, and it spread through newspapers, civic initiatives, and public education. When Chinese players competed against Western opponents, badminton matches became closely associated with narratives of national and cultural identity. The example of cross-sport athlete Kay Wong's participation across sporting contexts further illustrates that female athletic roles in China were more fluid. In badminton mixed doubles, she often occupied an auxiliary position, but in women's basketball, a sport more actively led by women, she assumed a more organizing and autonomous role.

The story of badminton's popularization in wartime Shanghai thus begins not with top-down policy or mass mobilization, but with the athletic excellence of a university-based team. The Flying Shuttles' victories galvanized media recognition, which in turn inspired organizational formation, spatial expansion, and state-society entanglement. Political regime change created new institutional openings that Chinese actors swiftly occupied, while the sport's rising profile reshaped everyday urban life. Through these intertwined forces, badminton evolved from an expatriate pastime into a citywide craze, propelled by champions, codified by federations, and claimed by the public.

Yet badminton did not fully realize the ideal of gender equality. Its mixed identity evolved into a practical compromise between two incompatible imperatives. On one side, mixed competition could be shown as a sign of gender equality and modern civility. On the other side, appeals to biological difference could be used to rationalize auxiliary roles for women in the name of efficient partnership and proper comportment. The division of labor in mixed doubles was packaged as natural and prudent, and in practice, it consolidated male authority over the long-rally, high-impact strokes that end points. The pattern is not one of exclusion. It is one of co-presence without co-power.

In the end, badminton's popularization was neither a simple democratization nor a straightforward path toward gender equality. Rather, it reflected a complex process of negotiation involving expatriate and Chinese players, elite and mass participants, and competing ideals of inclusion and protection. Its success lay in the sport's capacity to accommodate these tensions by offering a space that appeared modern and inclusive while preserving subtle forms of differentiation and hierarchy. To be at once modern and respectable, inclusive and differentiated, open and bounded. Accordingly, badminton in Shanghai shifted from a seasonal social supplement to a citywide mass sport, with the mixed-gender format serving as the public-facing structure that made universal participation seem attainable. The rise of badminton showed both sporting appeal and a practical way to meet the different roles expected of men and women, but also as evidence of a social formula that accommodated contradictory expectations.

This study has several limitations. First, it relies primarily on Chinese- and English-language newspapers published in China, which tend to reflect the perspectives of urban elites and Western-educated groups. Given the historical conditions of the Republican period, badminton was largely confined to urban spaces and specific social groups, making it difficult to directly capture the experiences of broader populations. Second, this study mainly focuses on Shanghai. As Shanghai developed organized badminton leagues earlier and generated more continuous media coverage, sources from other cities remain relatively limited, making it difficult to present a comprehensive national picture. Future research may address these limitations by incorporating a wider range of sources, examining developments in other cities, and exploring patterns of sports participation under different social conditions.

## Data Availability

The original contributions presented in the study are included in the article/Supplementary Material, further inquiries can be directed to the corresponding author.
